# Supplementary material for: Growth and feeding ecology of coniform conodonts
Source: PeerJ. 2021 Dec 14;9:e12505. doi: 10.7717/peerj.12505 (PMC8679908; doi:10.7717/peerj.12505)
Supplement: Supplemental Information 1 — Random effects of transect and element side, estimated for Sr/Ca values in the lamellar tissue of Proconodontus muelleri, with distance from the inside of the crown estimated as a fixed effect (n = 572). [file peerj-09-12505-s001.docx]

Table S1.

| **Level** | **Intercept** | **Slope** |
| --- | --- | --- |
| Transect 1 | 0.05207861 | -0.004786545 |
| Transect 2 | 0.05405914 | -0.006380387 |
| Transect 3 | 0.05133515 | 0.001721111 |
| Variance of the effect | 2.591×10^-6^ | 1.910×10^-5^ |
| Left | 0.05082564 | -0.002192981 |
| Right | 0.05397196 | -0.003998451 |
| Tip | 0.05267531 | -0.003254388 |
| Variance of the effect | 3.056×10^-6^ | 1.006×10^-6^ |
| Residual variance | 9.089×10^-6^ | |
