# Supplementary material for: Growth and feeding ecology of coniform conodonts
Source: PeerJ. 2021 Dec 14;9:e12505. doi: 10.7717/peerj.12505 (PMC8679908; doi:10.7717/peerj.12505)
Supplement: Supplemental Information 2 — Random effects of transect and element side, estimated for Sr/Ca values in the lamellar tissue of Pxanderodus equicostatus, with distance from the inside of the crown estimated as a fixed effect (n = 647). [file peerj-09-12505-s002.docx]

Table S2.

| **Level** | **Intercept** | **Slope** |
| --- | --- | --- |
| Transect 1 | 0.0568887 | 0.014622874 |
| Transect 2 |  | 0.013301909 |
| Transect 3 |  | 0.012390376 |
| Transect 4 |  | 0.017048164 |
| Transect 5 |  | 0.001594417 |
| Variance of the effect | 0.00 | 3.641x10^-5^ |
| Left | 0.05964813 | 0.004476287 |
| Right | 0.05412928 | 0.019106809 |
| Variance of the effect | 1.540×10^-5^ | 1.082×10^-4^ |
| Residual variance | 3.585×10^-6^ | |
