# Supplementary figures and images for: Growth and feeding ecology of coniform conodonts
Source: PeerJ. 2021 Dec 14;9:e12505. doi: 10.7717/peerj.12505 (PMC8679908; doi:10.7717/peerj.12505)

**A** *Pa.equicostatus*

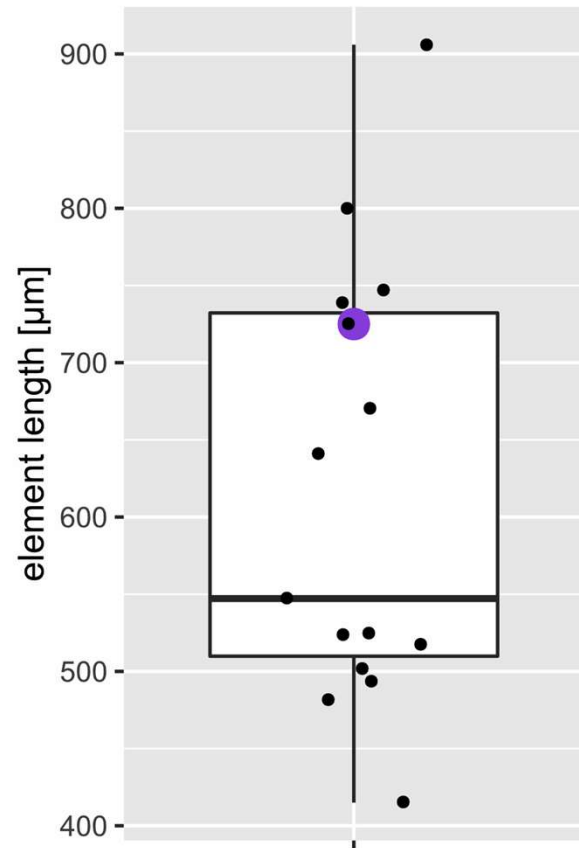

**B** *Pr.muelleri*

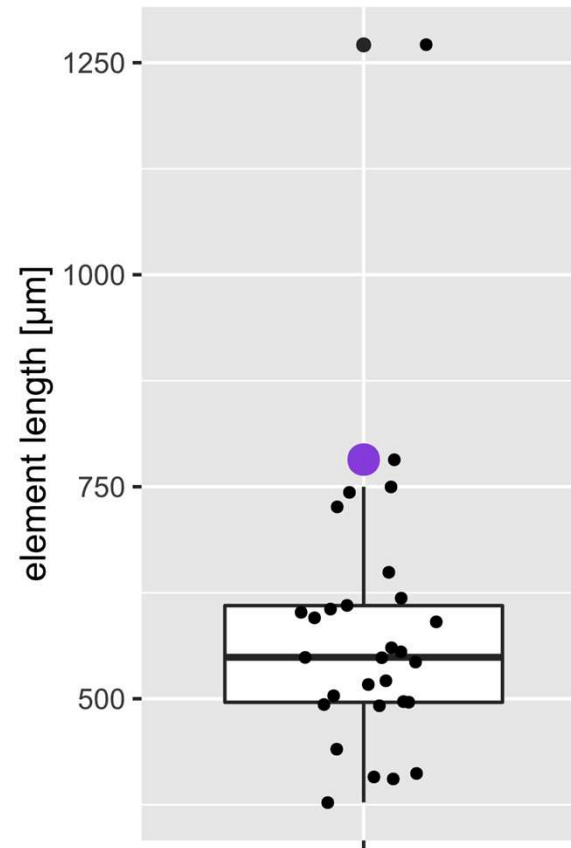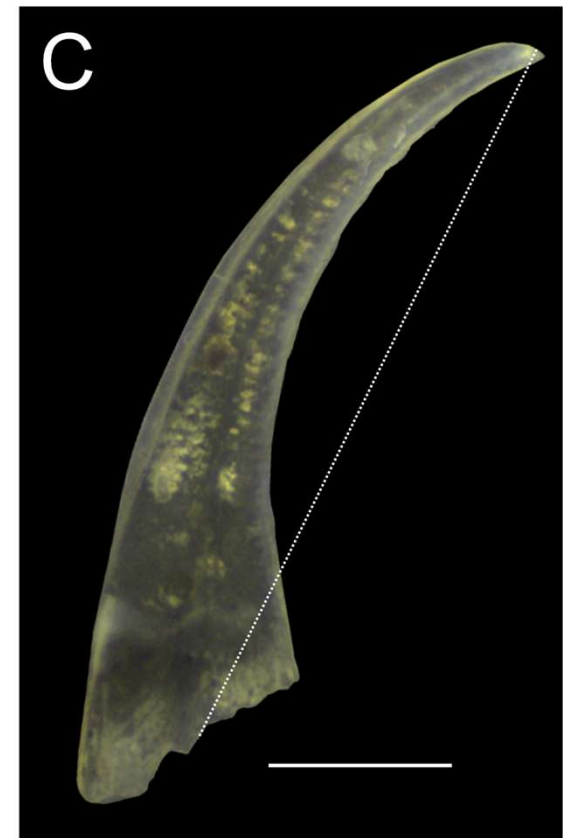

Supplement: Supplemental Information 3 — Relative distribution of the length of elements of Pa. equicostatus (A) and Pr. muelleri (B) from several literature sources (Percival & Zhen, 2006; Bagnoli & Stouge, 2014; Jarochowska et al., 2015; Jarochowska & Munneke, 2015; Dong & Zhan, 2017; Spiridonov et al., 2017). The marked dots represent the length of our elements (Pa. equicostatus 725 µm; Pr. muelleri 782 µm). C. Method of measuring element length on the example of a light microscope image of Pa. equicostatus with ImageJ (scale bar 200 µm). [file peerj-09-12505-s003.pdf]
