# Supplementary material for: Growth and feeding ecology of coniform conodonts
Source: PeerJ. 2021 Dec 14;9:e12505. doi: 10.7717/peerj.12505 (PMC8679908; doi:10.7717/peerj.12505)
Supplement: Supplemental Information 4 [file peerj-09-12505-s004.pdf]

GeoZentrum Nordbayern

FRIEDRICH-ALEXANDER UNIVERSITÄT ERLANGEN-NÜRNBERG
NATURWISSENSCHAFTLICHE FAKULTÄT

GeoZentrum Nordbayern • Loewenichstr. 28 • D-91054 Erlangen

GeoZentrum Nordbayern
Fachgruppe Paläoumwelt

Dr Emilia Jarochowska
Akademische Rätin auf Zeit

Loewenichstr. 28, 91054 Erlangen
Phone +49 9131 85-22967
Fax +49 9131 85-22690
Emilia.Jarochowska@fau.de

Erlangen, 2 August 2021

Specimen deposition and accession number

To whom it may concern,

I hereby confirm that the physical specimen of *Panderodus equicostatus* used in the study by Leonhard et al. submitted to PeerJ is stored as an SEM mount in the collections of GeoZentrum Nordbayern, Friedrich-Alexander-Universität Erlangen-Nürnberg, at Loewenichstr. 28, 91054 in Erlangen, Germany. It is available under the accession number EJ-12-V-19.25-001.

Kind regards,

Dr. Emilia Jarochowska
